# Polyphosphazenes as Adjuvants for Animal Vaccines and Other Medical Applications

**DOI:** 10.3389/fbioe.2021.625482

**Published:** 2021-03-04

**Authors:** Dylan J. Chand, Royford B. Magiri, Heather L. Wilson, George K. Mutwiri

**Affiliations:** ^1^Vaccinology & Immunotherapeutic Program, School of Public Health, University of Saskatchewan, Saskatoon, SK, Canada; ^2^Vaccine & Infectious Disease Organization-International Vaccine Centre (VIDO-InterVac), University of Saskatchewan, Saskatoon, SK, Canada; ^3^College of Agriculture, Fisheries and Forestry, Fiji National University, Nausori, Fiji

**Keywords:** adjuvants, polyphosphazenes, vaccines, immunity, animals, viruses, bacteria

## Abstract

Polyphosphazenes are a class of experimental adjuvants that have shown great versatility as vaccine adjuvants in many animal species ranging from laboratory rodents to large animal species. Their adjuvant activity has shown promising results with numerous viral and bacterial antigens, as well as with crude and purified antigens. Vaccines adjuvanted with polyphosphazenes can be delivered via systemic and mucosal administration including respiratory, oral, rectal, and intravaginal routes. Polyphosphazenes can be used in combination with other adjuvants, further enhancing immune responses to antigens. The mechanisms of action of polyphosphazenes have not fully been defined, but several systematic studies have suggested that they act primarily by activating innate immunity. In the present review, we will highlight progress in the development of polyphosphazenes as adjuvants in animals and their other medical applications.

## Introduction

Polyphosphazenes are high molecular weight, water-soluble, synthetic polymers that have been investigated for various biomedical applications including tissue regeneration and scaffolding, drug delivery, and stent and denture coatings. More recently, they have been extensively investigated as adjuvants to improve immune responses to vaccines. Vaccination continues to be a very important public health tool in the control of infectious diseases worldwide (Greenwood, 2014). Adjuvants are critical components of vaccines because they enhance antigen-specific immune responses that contribute to protection against disease. Subunit vaccines contain antigen components with varying degrees of purity. Highly purified antigens are poor immunogens and require the addition of adjuvants to generate protective immune responses. Polyphosphazenes are a relatively new class of adjuvants that enhance the magnitude, quality and duration of immune responses when co-administered with bacterial and viral antigens in several animal species including mice, pigs, sheep and cattle (McNeal et al., 1999; Andrianov et al., 2006, 2009, 2011; Mutwiri et al., 2007, 2008; Eng et al., 2010; Garlapati et al., 2011; Dar et al., 2012; Magiri et al., 2018). The two most investigated polyphosphazenes are poly [di(carboxylatophenoxy)phosphazene] (PCPP) and poly[di(sodiumcarboxylatoethylphenoxy)phosphazene] (PCEP) (Mutwiri and Babiuk, 2009). Changes in the synthesis and formulation as a soluble adjuvant or microparticle impacts how they influence immune responses (Andrianov et al., 2004). PCEP has been shown to significantly increase IgG1 and IgG2a antibody production compared to PCPP (Mutwiri et al., 2008) and also to induce 1,000-fold higher antibody titers compared to alum when co-administered subcutaneously with an influenza antigen in mice (Mutwiri et al., 2007). Relative to PCPP, PCEP also promotes a stronger mixed Th1/Th2-type T cell response leading to broad spectrum immunity (Mutwiri et al., 2007).

## Polyphosphazenes

### Chemical Composition and Structure

**Figure 1:**
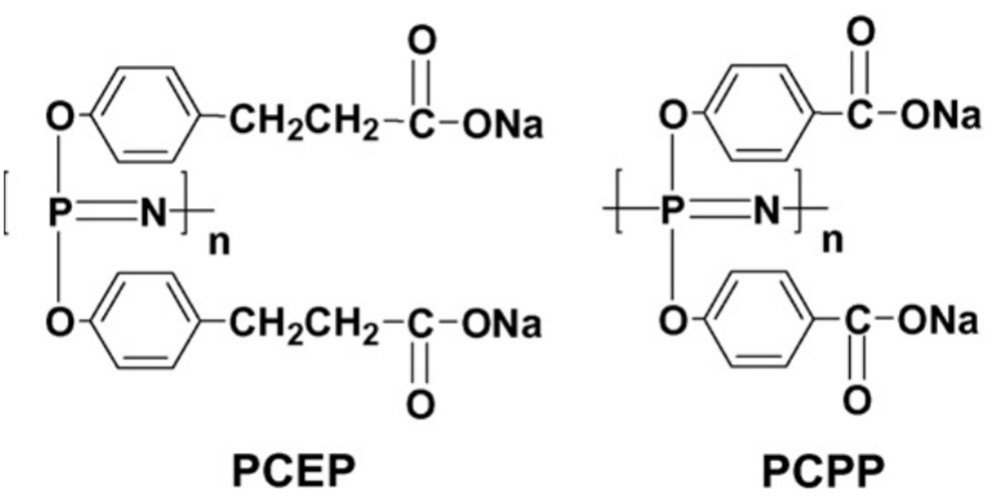
Chemical structures of the polyphosphazene polymers PCEP and PCPP *(reprinted from Mutwiri et al.*, *2007**, copyright 2007, with permission from Elsevier)*.

Polyphosphazenes are synthetic polymers that consist of a backbone with alternating phosphorus and nitrogen atoms and organic side groups attached to the phosphorus (Payne et al., 1998). The structures of the polyphosphazene molecules has been altered to incorporate ionic moieties resulting in water soluble salts. This, along with the potential hydrolytic degradability of their main chain make them attractive candidates for vaccine adjuvants and delivery systems. In this regard, two of the most investigated polyphosphazene adjuvants are PCEP and PCPP shown in Figure 1.

### Synthesis Methods

The synthesis of PCEP was described in a previous publication (Mutwiri et al., 2007) as follows: PCEP was prepared by reacting a solution of sodium salt of methyl 3-(4-oxyphenyl) propionate with polydichlorophosphazene in diglyme at 120°C for 10 h. The resulting polymer, containing ester groups in side chains was hydrolyzed using aqueous potassium hydroxide at 85°C. Polymer was recovered by precipitating in ethanol and additionally purified by precipitating in sodium chloride solution and then in ethanol in its salt form. The high molecular weight nature of the polymer was confirmed by gel permeation chromatography, which was configured as follows: Waters 600 HPLC pump, Waters 717 plus Autosampler, an Ultra- hydrogel Linear column, a multi-angle laser light scattering (MALLS) detector (DAWNDSP-F, Wyatt Technology, Santa Barbara, CA), a Waters 996 Photo Diode Array detector, and a Waters 410 refractive index detector (Waters, Milford, MA). Phosphate buffered saline (PBS, pH 7.4) containing 5% acetonitrile was used as a mobile phase. Polyphosphazene adjuvants PCPP and PCEP were designed and synthesized by Parallel Solutions Inc. (Cambridge, MA). Aqueous solutions of both polymers were stored at room temperature in the dark and were found to retain activity for over several months under these storage conditions. Batches of polyphosphazenes were tested and found to have endotoxin levels that were below 0.034 ng/ml as assessed by Limulus Amebocyte Lysate assay (Biowhittaker, Walkersville, MD, USA). The synthesis of PCPP was described previously [13] and will not be repeated here.

### Preparation of Antigen-Polyphosphazene Formulations

One of the most attractive features of polyphosphazene adjuvant is the simplicity with which an antigen-adjuvant formulation can be prepared. These polyphosphazenes are water soluble and can be simply mixed with antigen solutions at room temperature. Antigens spontaneously self-assemble with the polyphosphazenes into complexes predominantly formed through electrostatic interactions between negatively charged polyphosphazene and positively charged antigens, and stabilization of complexes is achieved through formation of hydrogen bonds and hydrophobic interactions (Andrianov et al., 2016). Details of interactions between polyphosphazenes and various antigens and the sizes of the resulting antigen-polyphosphazene complexes (generally 70–150 nm in diameter) have been reported in a recent review by Andrianov and colleagues (Andrianov and Langer, 2021).

## Mechanisms of Action of Polyphosphazene Adjuvants

**Figure 2:**
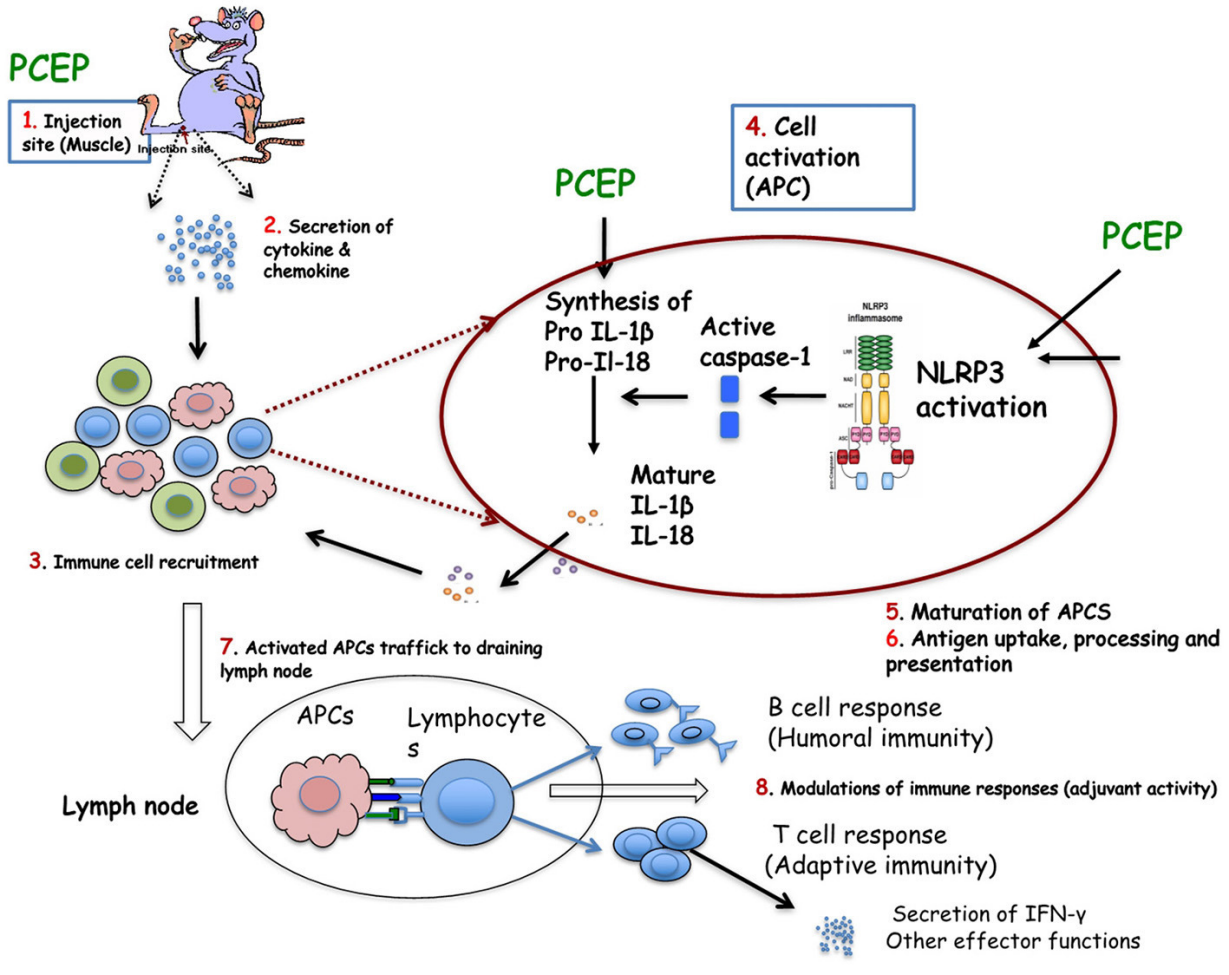
Proposed mechanism of action of PCEP: (1) PCEP injected into muscle is transiently retained locally, induces genes involved in innate immunity. (2) PCEP induces local cells to secret cytokines and chemokines. (3) The secreted cytokines and chemokines initiate recruitment of various immune cells, including APCs (such as DCs) to the injection site. The recruited cells are activated and secrete more cytokines and chemokines, which in turn chemoattracts other immune cells. All these events lead to formation of local immunocompetent environment at the injection site. (4) Recruited DCs (which express various PRRs both on the surface (TLRs, CLRs) and intracellularly (NLRs and RLRs) are recognized and/or activated by PCEP resulting in activation of inflammasome. It is not yet known whether PCEP modulates (5) Antigen uptake, process and presentation, or (6) maturation of APCs. (7) PCEP increases the trafficking of APCs to the draining lymph nodes to interact with antigen-specific B or T cell to (8) Resulting in potent antibody secreting B cells and/or effector CD8+ T cell responses. These events constitute adjuvant activity.

The MOA of PCEP or PCPP has not been fully elucidated. Early studies suggested that PCPP did not act by depot effect, since excision of injection sites in mice had no effect on the ensuing immune responses (Payne et al., 1998). While there are still gaps in our understanding of its MOA, we have gained significant knowledge on how PCEP interacts with the immune system to enhance antigen-specific immune responses. Most of the information on the MOA of polyphosphazene adjuvants has been from studies in which animals were injected with antigen alone, in the absence of antigen (Supplementary Table 2). Based on evidence from several MOA studies in mice, we have proposed a model to explain the MOA of PCEP, which is summarized in a diagram (Figure 2). Evidence in support of this model is provided below under five (5) subheadings.

### Upregulation of Cytokines and Chemokines

IM injection of mice with PCEP induced gene expression of many genes associated several adjuvants including chemokine genes CCL-2, CCL-4, CCL-5, CCL-12 and CXCL-10 in mice (Awate et al., 2012). Additionally, a major transcription factor NF-*k*B gene and the inflammatory cytokine TNF-α gene were also up-regulated in response to PCEP in mice (Awate et al., 2012). At the protein level, PCEP promoted significant local production of Th1-type proinflammatory cytokines (IL-1β, Il-6, IL-18 IFN-γ and TNF-α) and Th2-type cytokines (IL-4 and monocyte chemo-attractants CCL-2 and CXCL-10) at the site of injection in mice but not systemically (Awate et al., 2012).

In pigs, PCEP induced significant production of interleukin IL-1β and IL-13 at the site of injection and IL-1β and IL-6 at the draining lymph nodes. Emulsigen (a commercially available oil-in-water emulsion adjuvant for animal vaccines) promoted the production of IL-1β, IL-6, and IL-12 at the site of injection but not in the draining lymph nodes (Magiri et al., 2019).

### Cell Recruitment to the Site of Injection

PCEP have been shown to induce recruitment of immune cells to the site of injection in mice. This local cell recruitment was presumably mediated by the several chemokines released as noted in Upregulation of Cytokines and Chemokines above. The recruited cells peaked within a week and were predominantly neutrophils, but also macrophages, CD4^+^ T cells, CD8^+^ T cells and CD19^+^ B cells, monocytes and DCs to the injection site (Awate et al., 2014b). The numbers of recruited cells were significantly higher in mice immunized with PCEP adjuvanted vaccines than in the alum-adjuvanted vaccines. Confocal analysis revealed that many recruited myeloid cells (but only a few lymphocytes) showed evidence of intracytoplasmic lysosomal localization of PCEP (Awate et al., 2014b) which PCEP potentiates immune responses.

In pigs, PCEP induced recruitment of macrophages, T and B cells, leucocytes and necrotic debris at the site of injection (Magiri et al., 2019).

### Activation and Maturation of Dendritic Cells

In *in vitro* studies, PCEP activated the NLRP3 inflammasome in a Caspase 1-depedent manner which led to the processing of interleukin IL-1β, IL-18 and IL-33 stimulated splenic DCs in mice (Awate et al., 2014a). However, while inflammasome activation may contribute to adjuvant activity by promoting a pro-inflammatory environment at the site of injection, it is yet to be confirmed whether inflammasome activation is required for the adjuvant activity of PCEP. Interestingly, PCEP injection in mice increased the expression of TLR4 and TLR9 at the site of injection (Awate et al., 2012). The biologic significance of this observation is unclear, given that PCEP is not of microbial origin. PCEP has strong avidity for TLR7, TLR8, and TLR9 (Sasai and Yamamoto, 2013; Andrianov et al., 2016). Furthermore, direct activation of immune cells by PCPP and PCEP through TLR signaling pathway, both on the external cell surface (TLR4) and endosome (TLR3 and TLR9) receptors has been reported (Reed et al., 2013; Sasai and Yamamoto, 2013). It was reported that polyphosphazene adjuvants also induced the maturation of DCs *in vitro* (Andrianov et al., 2006, 2016).

### Antigen Uptake, Processing and Presentation

*In vitro* studies suggested that PCPP promoted the uptake of Gag antigen of HIV, increased expression of co-stimulatory molecules in DCs and enhanced Gag antigen presentation to CD4+ T cells (Palmer et al., 2014). In a separate study, it was reported that PCEP, but not PCPP, disrupted early endosomes, suggesting that PCEP may promote cross-presentation of antigen (Andrianov et al., 2016). If this is the case, then PCEP promotes the ability of DCs to take up, process, and present extracellular antigens with MHC class I molecules to CD8 T cells, a pathway critical for the development of Th1 responses. This response may explain, at least in part, why PCEP induces a mixed Th1/Th2 while PCPP induces predominantly Th2 and no Th1 type immune responses.

### Trafficking of Activated Cells to the Draining Lymph Nodes

IM injection of mice with PCEP resulted in increased numbers of myeloid cells and lymphocytes in draining lymph nodes (Awate et al., 2014b). Neutrophils and DCs were detected in the lymph nodes as early as 3 h after injection, while macrophages followed in the next 24 h. Interestingly, lymphocytes (mainly CD4+ and CD8+ T cells) were also increased in the lymph nodes after injection of PCEP (Awate et al., 2014b). All the cells types were significantly higher in mice injected with PCEP compared to those injected with alum. In pigs, PCEP induced infiltration of leucocytes, resulting in a local inflammatory response in the draining lymph nodes (Magiri et al., 2019).

Therefore, the MOA of PCEP involves early activation innate immune responses that involves cell recruitment to site of injection cell activation promoting a strong immunostimulatory environment at the site of injection. and then trafficking of activated cells to the draining lymph nodes.

## Application of Polyphosphazenes as Vaccine Adjuvants in Various Animal Species

### Viral Antigens

**Figure 3:**
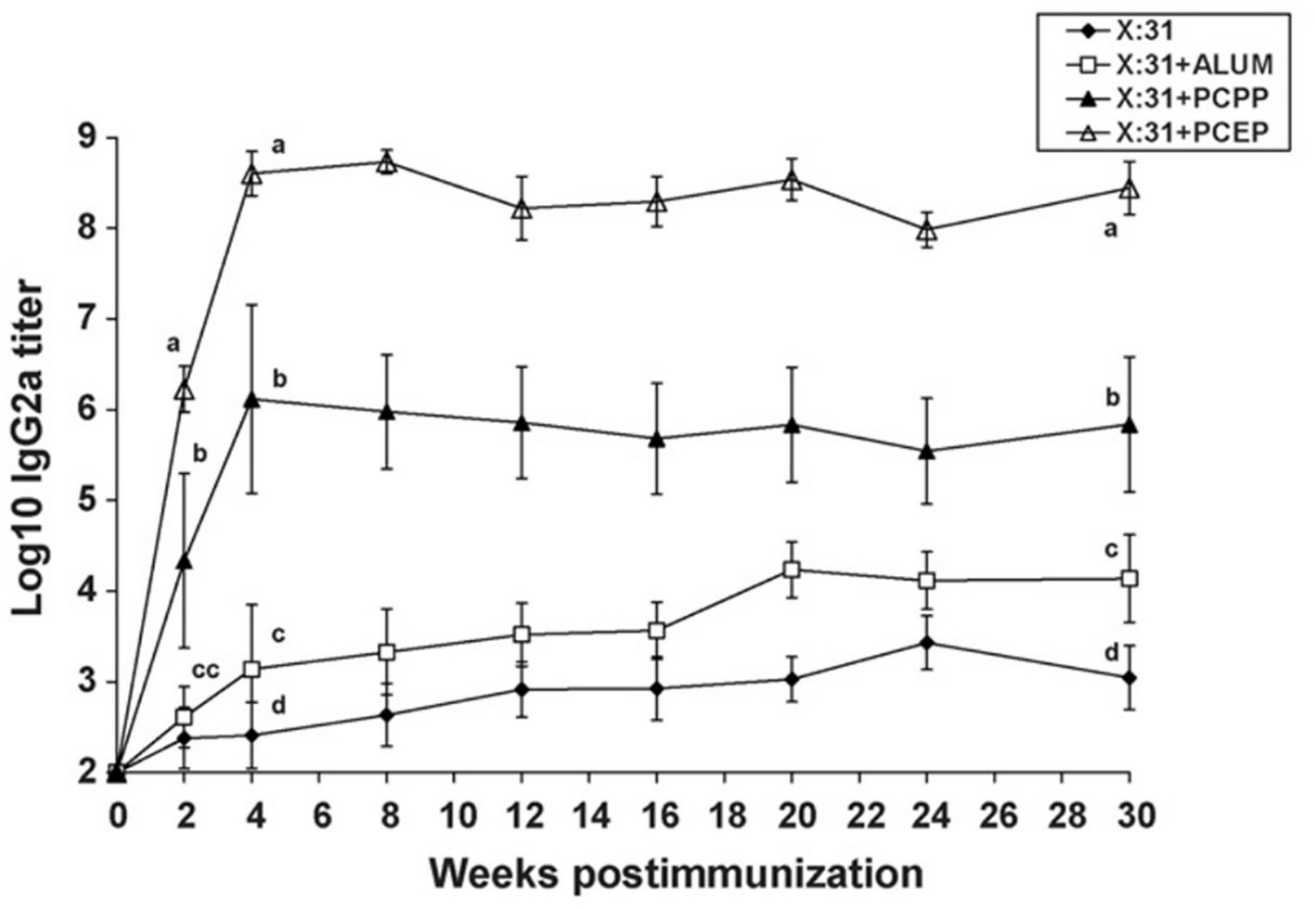
Immune responses to low dose of influenza virus X:31 antigen: IgG2a antibody responses were assayed in serum of BALB/c mice (*n* = 5 per group) given a single s.c. immunization with a low dose (0.2_g) of X:31 antigen alone, X:31 + alum, X:31 + PCPP or X:31 + PCEP. Each data point represents mean ± S.E.M. for titers as determined by ELISA. Groups with different letters are significantly different from each other (*p* < 0.05). Data are representative of two independent experiments *(reprinted from Mutwiri et al.*, *2007**, Copyright 2007, with permission from Elsevier)*.

Polyphosphazenes have been used as experimental adjuvants in a variety of viral antigens. Immunization of mice with influenza virus X:31 antigen co-formulated in PCPP increased IgG titers upon subcutaneous (SC) immunization in mice (Andrianov et al., 2004; Mutwiri et al., 2007). When compared to the conventional adjuvant alum, PCPP and PCEP both induced antibody titers that were 100-1,000-fold higher than alum (Figure 3). The differences between the three adjuvants were more dramatic when IgG2a titers were examined. PCEP induced IgG2a titers that were 100-fold higher than PCPP and several orders of magnitude (more than 10,000-fold) higher than alum (Figure 1). Furthermore, PCEP promoted both IgG1 and IgG2a titers (indicative of a mixed Th1/Th2 type immune response), while both PCPP and alum promoted a Th2 type but no Th1, as indicated by poor IgG2a antibody response (Mutwiri et al., 2007). In addition, PCEP required only 1/5 of the dose of X:31 antigen to induce antibody titers similar to alum (Figure 3). Thus, PCEP has the potential to reduce the cost of vaccination by reducing the number of vaccinations and since only minimal doses of antigen are required to induce significant immunity, the cost of vaccination could be dramatically reduced. In a separate study influenza, PR8 antigen of influenza Virus H1N1 adjuvanted with PCPP increased mucosal IgA, enhanced IgG1 and IgG2a antibody responses, induced a mixed Th1/Th2 cytokine responses which contributed to long term B cell memory response following both systemic and mucosal immunization in mice (Shim et al., 2010). Furthermore, the immune responses induced were protective against lethal H1N1 challenge (Shim et al., 2010). In ferrets, PCPP adjuvanted influenza vaccine HA H5N1 demonstrated dose sparring, increased vaccine stability, and an increased survival rate in lethally challenged animals (Andrianov et al., 2011).

Coformulation of the viral antigens such as HBsAg, PR8, or influenza X:31 with PCEP resulted in significant increases in both IgG1 and IgG2a antibody responses in mice (Mutwiri et al., 2007, 2008; Shim et al., 2010). Recombinant MV-H (Measles Virus recombinant “H”) protein of measles virus adjuvanted with PCEP induced significant IgG1 and IgG2a antibody responses and neutralization titers *in vivo* (Lobanova et al., 2012). *In vitro*, increased MV-H-specific IFN-γ and IL-5 was observed in the splenocytes indicating strong cell mediated immunity (Lobanova et al., 2012). Further, the recombinant protein's immunogenicity was retained 2 weeks after storage, suggesting the vaccine formulation was stable (Andrianov et al., 2011; Lobanova et al., 2012). PCEP efficiently promoted a balanced Th1/Th2 type response, following both mucosal and systemic immunization, indicating that PCEP is versatile as an adjuvant (Mutwiri et al., 2007; Eng et al., 2010).

In recent studies in pigs, PCEP was shown to be an effective adjuvant with an experimental swine influenza vaccine. PCEP promoted strong immune responses and protection against homologous swine influenza H1N1 virus but failed to cross-protect against the heterologous H3N2 virus (Magiri et al., 2018, 2020). A vaccine with PCEP administered intradermally was superior to that injected intramuscularly (IM), suggesting the importance of route of administration in inducing protective immunity (Magiri et al., 2020). Respiratory syncytial virus (RSV-F), HN of parainfluenza virus 3 and inclusion body hepatitis virus (IBHV) antigens have also been used with polyphosphazene adjuvants, but these are discussed under combination adjuvants further below in this review.

### Bacterial and Other Types of Antigens

Polyphosphazene adjuvants have also been used with bacterial antigens. Pertussis toxoid (PTd) antigen elicits a Th-1 biased response when adjuvanted with PCEP (Gracia et al., 2011), or even multiple adjuvants such as a triple adjuvant formula like PCEP+CpG+IDR when given by IN administration in mice (Garlapati et al., 2011). Subcutaneous injection of pigs with an *Actinobacillus pleuorpnemoniae* (APP) outer membrane antigen (OmlA) adjuvanted with PCEP showed significantly increased IgG1 and IgG2a antibody responses and also increased IFN-γ when compared to pigs vaccinated with OmlA adjuvanted with the commercial adjuvant, Emulsigen (Dar et al., 2012).

A large proportion of studies performed using polyphosphazenes as adjuvants have been centered around academic antigens such as ovalbumin (OVA). Because these antigens alone do not induce strong immune responses, they are often used alongside adjuvant formulations such as the triple adjuvant formulation (Garlapati et al., 2011) to better relay the abilities of the adjuvant in relation to an antigen. Studies involving academic antigens Bovine Serum Albumin (BSA) with PCPP in mice promoted increased serum IgG, however such titers were dependent on the structure of the PCPP backbone (Andrianov et al., 2005). PCPP administered with OVA promoted increased IgG1 and IgG2a antibody response and ultimately a predominantly Th1 response (Garlapati et al., 2010), findings which have been further corroborated by more recent studies involving PCEP as a combination adjuvant. Oral administration of PCEP with OVA induced significant anti-OVA specific IgM and IgG, alongside enhanced IgG1 and IgG2a serum antibodies in pigs (Pasternak et al., 2014).

### Comparison of Routes of Administration

Polyphosphazene-adjuvanted vaccines have been tested in a variety of routes including IN, SC, intrauterine (IU), Oral, and intrarectal (IR) with various antigens and in different animal models and in humans (Supplementary Table 1). However, parenteral immunization has been the predominant method of vaccination for animals (Zhang et al., 2015). Historically, subcutaneous injection has been the most commonly used method as it represents the route of application of many animal vaccines to date (Cook, 2008).

In a study evaluating various routes of administration of influenza virus X:31 antigen, IN and SC had superior adjuvant activity compared to the Oral and IR routes; significantly higher IgG1 titers and increased IgG and IgA in nasal secretions yielded results conferring systemic and humoral response not seen with IR or Oral vaccination (Eng et al., 2010). Intranasal (IN) immunization with PCEP+X:31 induced significantly higher IgA titers in all mucosal secretions (nasal, lung, vaginal) compared to the other routes, particularly the IR route which showed negligible immunogenicity (Eng et al., 2010).

While IN and SC have proven to have more optimal response than Oral or IR, the distinction between the two is also significant. In a study where IN immunization was compared to SC, there were distinct differences in antibody response, neutralizing titers, and viral replication and protection against challenge. A two-dose study comparing IN/IN, IN/SC, SC/IN, and SC/SC, revealed that two IN immunizations (IN/IN) were the most effective in inducing increased IgG1 and IgG2a, as well as reducing viral replication in the lungs and conferring protection in experimentally infected mice (Mapletoft et al., 2010).

Intradermal (ID) immunization outperforming IM injection in pigs is a theme documented by a 2018 study comparing ID and IM with swine influenza virus (SIV) antigen. PCEP adjuvanted pigs injected ID displayed greater induction of humoral immunity than IM injected pigs (Magiri et al., 2018). Furthermore, the ID vaccinated group also developed greater neutralizing titers compared to IM (Magiri et al., 2018) hence ID was proven to be the superior route in pigs. The superior performance of the ID route is presumably due to the presence of professional antigen presenting cells (APCs) such as dendritic cells (DC) are more frequent in the skin relative to muscle tissue.

In another study in pigs, it was noted that when Hepatitis B Surface Antigen (HBsAg) was injected alongside PCPP, ID vaccination yielded 10-times higher IgG titers; and also induced significantly higher HBsAg-specific titers than IM injection (Andrianov et al., 2009). ID injection of HBsAg adjuvanted with PCPP displayed a significant dose sparring effect, where a 10 μg ID dose induced 10-fold higher antibody titer than 20 μg of the antigen alone (Andrianov et al., 2009). The significance of PCPP as a part of vaccine formulation was also confirmed by 100-fold increase in antibody titers for the polyphosphazene adjuvanted microneedle system over the ID injection of non-adjuvanted antigen (Andrianov et al., 2009).

Intrauterine vaccination has produced mixed results between pigs and rabbit species as it pertains to the use of Tri-Adj formulations containing PCEP. In pigs, inactivated PPV and PEDV+FliC antigens formulated with Tri-Adj failed to elicit a humoral immune response, despite the adjuvant's ability to directly stimulate chemokines CCL2, IFN-β, and CCL4 in uterine epithelial cells (UECs) (Hamonic et al., 2020). In rabbits, when Tri-Adj formulation PCEP+poly:I:C+HDP was administered with three different antigens, OVA, tGD, and vP2-TrX, IM and IU routes both demonstrated increased IgG and IgA serum titers for OVA and tGD antigens (Pasternak et al., 2017). However, in this same experiment it became apparent that the IU route was not as effective in stimulating systemic immunity, as indicated by increased IgG only via the IM route for the rVP2-TrX antigen (Pasternak et al., 2017).

## Combination of Polyphosphazenes With Other Adjuvants

### Why Use Combinations of Adjuvants?

Historically, vaccines have employed a model of “one adjuvant, one vaccine,” mainly because of regulatory hurdles on safety and/or for purely economic reasons. It is unlikely that a single adjuvant will be able to fulfill all the required immunologic properties in the many different vaccines. For example, alum is known to only stimulate Th2 immune cells leading to increased production of antigen-specific antibodies but is poor or even inhibitory to Th1 or cytotoxic responses. This limitation in single adjuvants can be alleviated by the development of adjuvant combinations such as MF59, AS03, or AS04 that seek to further strengthen antigen-specific humoral (Th2) responses and also broaden responses by increasing Th1 or cytotoxic responses. Evidence has been accumulating over the last two decades that formulation of a vaccine with multiple adjuvants may act synergistically to elicit dramatic increases in mixed Th1 and Th2 types of immune responses (Kindrachuk et al., 2009; Mutwiri et al., 2011; Salvador et al., 2012; Mount et al., 2013; Levast et al., 2014; Ciabattini et al., 2016; Didierlaurent et al., 2017; Madan-Lala et al., 2017). Furthermore, different adjuvants are required to stimulate immune responses required following different vaccination strategies taking into account the pathogen, the type of antigen, the immune status and age of the vaccine and the application route in order to improve vaccine efficacy (Garçon and Di Pasquale, 2017). Most importantly, the stimulation of a cell-mediated Th1 response and cytotoxic T lymphocytes (CTLs) is highly sought since actual vaccines antigen mostly stimulate predominantly humoral (Th2) immune responses. Combination adjuvants are particularly used to enhance and/or direct the immune responses toward a Th1-, Th2-, or Th17-type responses (Kindrachuk et al., 2009; Salvador et al., 2012; Levast et al., 2014). Adjuvants target different pattern-recognition receptors (PRR), both endosomal and intracellular. Their combinations based on these targets can enhance antigen-specific humoral and cellular immune responses (Gutjahr et al., 2016). For instance, Toll-like receptor (TLR) agonist MPL A adsorbed on aluminum salts (AS04TM, GlaxoSmithKline) in a combination results in the stimulation of increased production of antigen-specific antibodies and an enhanced cell-mediated response by causing a local and temporary cytokine response (Reed et al., 2009; Garcon et al., 2011; Del Giudice et al., 2018). Due to the short half-life of most immunostimulatory adjuvants *in vivo*, combining a delivery vehicle adjuvant with an immunostimulatory adjuvant like polyphosphazene may increase the magnitude and modulate the quality of immune responses (Weiner et al., 1997).

### Examples of Combination Adjuvants Containing Polyphosphazenes

**Figure 4:**
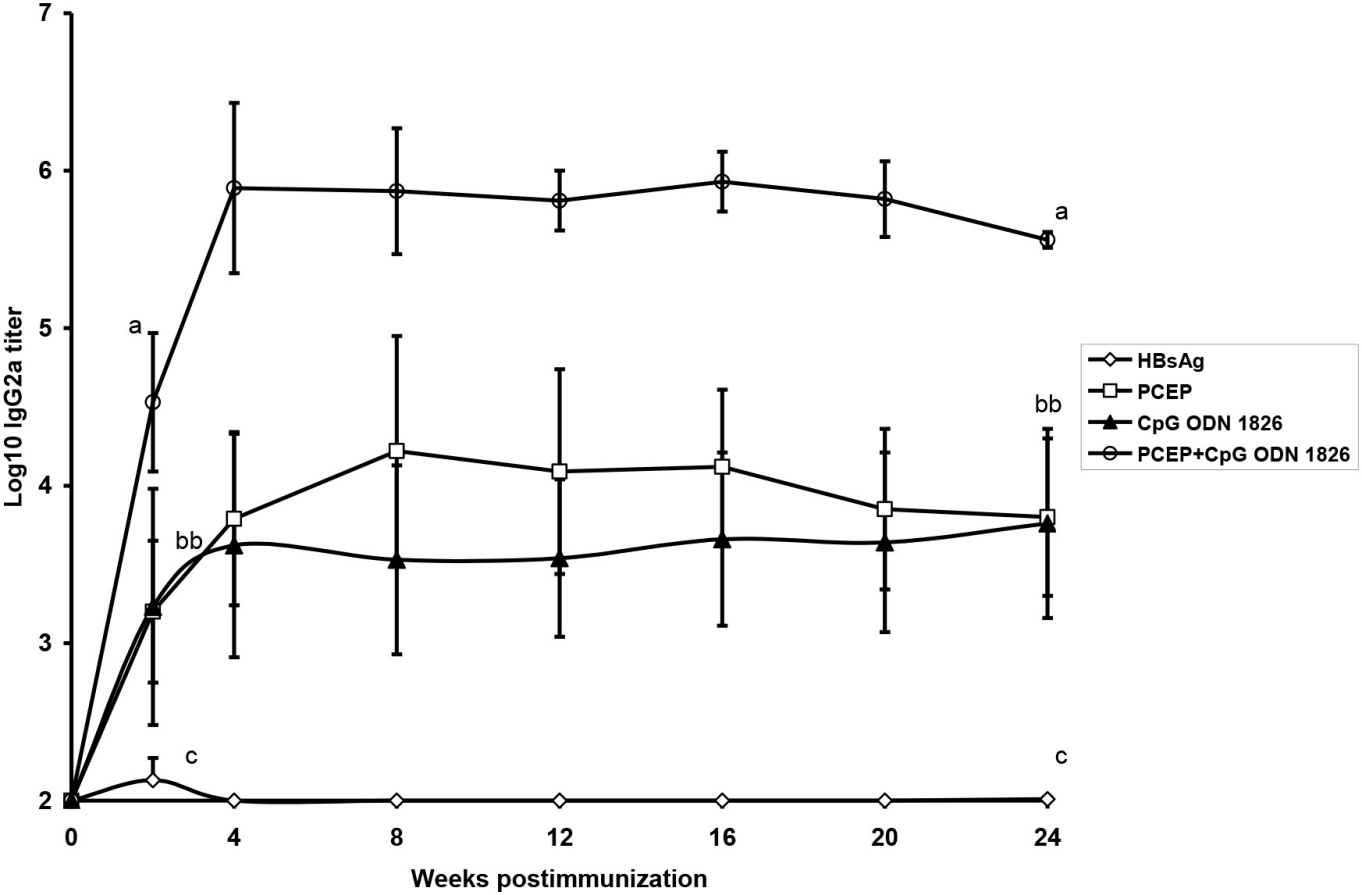
Serum HBsAg-specific IgG2a antibody titers in mice given a single SC immunization with a dose of 0.2 _g of HBsAg alone, HBsAg + PCEP, HBsAg + CpG or HBsAg + PCEP + CpG. Each data point represents mean ± S.E.M. of titers of anti-HBsAg as determined by ELISA. Groups with different letters are significantly different (*p* < 0.05) *(reprinted from Mutwiri et al.*, *2008**, Copyright 2007, with permission from Elsevier)*.

As demonstrated with numerous other adjuvants, the adjuvant potential of polyphosphazenes can be greatly enhanced by combining with other adjuvants. Subcutaneous immunization of mice with Hepatitis B surface antigen (HBsAg) plus PCEP or PCPP combined with the TLR agonist CpG ODN resulted in enhanced production of HBsAg-specific antibody responses compared with mice immunized with HBsAg plus any of the three adjuvants when used alone (Mutwiri et al., 2008). When IgG2a antibody titers were compared, CpG ODN and PCEP both induced comparable titers, but the combination of PCEP+CpG ODN produced ~100-fold higher titers than the individual adjuvants (Figure 3). The titers were maintained for almost 6 months (Figure 4). Similarly, immunization of mice with PCPP microparticles encapsulating OVA and CpG ODN generated higher antigen-specific antibody responses compared to antigen alone (Garlapati et al., 2010; Wilson et al., 2010).

In several studies it has been demonstrated that the use of polyphosphazenes in triple adjuvant combination (TriAdj) consisting of PCEP or PCPP plus TLR agonist (CpG or poly:I:C) and Host Defense Peptide (HDP) induced robust immune responses with various antigens, in multiple species and in diverse routes of delivery. Similar observations were made in a separate study, mice immunized with OVA plus TriAdj combination significantly enhanced antibody and cell mediated immune responses via both MHC class-I and MHC class-II pathways, promoting a more balanced Th1/Th2 immune response, compared to mice immunized with OVA plus any of the individual adjuvants (Kovacs-Nolan et al., 2009a). Further, S.C. immunization of mice with *Bordetella pertussis* antigen plus TriAdj reduced bacterial load after challenge and increased antigen-specific IL-17 secreting cells relative to vaccine containing only one or two of the other adjuvants (Garlapati et al., 2011). Furthermore, TriAdj formulation with PTd increased IgG1 responses in adult mice in addition to superior serum IgG2a antibody titers in both adult and neonatal mice compared to mice immunized with each adjuvants alone (Gracia et al., 2011). Additionally, recombinant truncated bovine respiratory syncytial virus (bRSV) fusion protein (Δ-F) plus TriAdj enhanced secretion of antigen-specific serum antibody titres compared with mice immunized with antigen alone (Kovacs-Nolan et al., 2009c).

IN vaccination with a formalin-inactivated bRSV antigen plus TriAdj induced systemic and mucosal immunity in mice (Mapletoft et al., 2010) which then significantly reduced viral replication upon virulent bRSV virus challenge in mice (Mapletoft et al., 2008). OVA antigen has also been formulated with lipidic (L) Tri-Adj, and microparticle formulated (MP) Tri-Adj and given intranasally in mice. These formulations all resulted in balanced Th1/Th2 responses, but with L-Tri-Adj being the most effective and inducing significantly greater levels of IgG1 titers than the other formulations, as well as enhanced IgA response (Wasan et al., 2019). L-Tri-Adj and MP Tri-Adj both also promoted increased IgG2a and IFN- γ from lymphocytes indicating a stronger CMI than that of Tri-Adj alone (Wasan et al., 2019).

Subcutaneous immunization of cattle on days 0 and 90 with hen egg lysozyme (HEL) antigen adjuvanted with TriAdj showed superior antigen-specific humoral responses and cell-mediated immune responses relative to immunization with antigen alone (Kovacs-Nolan et al., 2009b). Purified E2 antigen of BVDV-2 (Bovine viral diarrhea virus) formulated in TriAdj and administered IM significantly increased E2-specific VN antibodies along with IFN-γ, CD4+/CD8+ T cells, indicative of a mixed Th1/Th-2 type immune responses (Snider et al., 2014). The purified E2 antigen also stimulated significant protection against virulent BVDV-2 4 weeks after vaccination; compared to the control PBS-immunized cattle, those immunized with the E2 vaccine had lower body temperatures, significantly less weight loss, and significantly higher WBC counts than the PBS treated cows during days 6–12 of the trial (Snider et al., 2014). Furthermore, increased expression of the CD25+ marker which indicated the presence of CTLs was also detected and with the exception of a single animal, vaccinated calves did not show evidence of viral replication (Snider et al., 2014). A similar robust antibody response was also noted in an ID immunization with the same adjuvant and antigen formulation where APCs and DCs were significantly increased in number upon challenge with no clinical sickness, leukopenia, or viral shedding observed (Sadat et al., 2017).

Respiratory syncytial virus F (RSV-F) protein formulated with Tri-Adj (CpG ODN+PCEP+HDP) induced a robust immune response in mice immunized via IN route. Mucosal and systemic immunity was effectively induced as indicated by the overall antibody responses including IgA, IgG1, and IgG2a (Garg et al., 2014, 2016, 2017, 2019; Sarkar and Garg, 2016). Significant antigen-specific CD8+T cell production was also observed (Garg et al., 2014, 2017), as well as reduction in viral replication in the lungs (Sarkar and Garg, 2016; Garg et al., 2017). Furthermore, interferon secretion, monocyte and DC trafficking to lymph nodes and recruitment were also observed with this formulation (Garg et al., 2014; Sarkar and Garg, 2016). This experimental vaccine formulation activated prolonged immunity in both arms of adaptive immunity and ultimately provided protection against a virulent RSV challenge (Garg et al., 2017). In a separate study, RSV-F and HN of para-influenza virus 3 (PIV3) antigens administered IM adjuvanted with Tri-Adj resulted in more efficient transfer of maternal antibodies from ewes to newborn lambs (Garg et al., 2019).

In rabbits, IM or IU immunization with a single dose of OVA, truncated glycoprotein D (tGD) from bovine herpesvirus, and a fusion protein of porcine parvovirus protein VP2 and bacterial thioredoxin (rVP2-TrX) formulated with TriAdj induced antigen-specific antibody responses systemically (serum) and within the local (uterus) and distal mucosa (lungs and vagina) (Pasternak et al., 2017). Thus, PZ as part of the TriAdj combination dramatically enhanced the magnitude of immune responses in variety of viral and bacterial antigens resulting in balanced immunity for broader protection.

Chickens have also been the subject of studies in combination adjuvant studies. Eighteen-day-old embryonating eggs were inoculated with PCPP/PCEP+CpG ODN, and 4 days after exposure to the adjuvants or 1 day post hatch, they were inoculated SC with *E. coli* (Taghavi et al., 2009). No adverse reactions or impacts on hatchability were observed, along with significant decrease in mortality in embryos immunized compared to the control group (Taghavi et al., 2009). Birds that received CpG ODN + PCEP had a significantly higher survival rate compared to the other groups (55%), and relative risk of mortality was significantly reduced in PCPP (0.25) and PCEP (0.33) formulations as well (Taghavi et al., 2009). In a separate study, immunization of chickens with inactivated IBHV vaccine formulated in PCEP with avian beta defensin (ABD) induced significant increases in IgG antibody response, IFN-γ, IL-12 (p40), IL-6, and overall a balanced Th1/Th2 immune response (Dar et al., 2015).

## Discussion

In the last few decades, there has been increased interest in research on adjuvants leading to identification of numerous substances with adjuvant potential. Some of these new adjuvants have undergone pre-clinical development, but few ever make it into clinical trials, and even fewer ever become used in commercial vaccines.

There are several challenges to using new adjuvants in commercial vaccines, and these challenges go beyond proof of efficacy. Most, if not all, vaccine companies have their own adjuvants. One of the widely known portfolio of adjuvants is GSK's (Glaxo Smith Kline) adjuvant system (AS), which has relatively new adjuvants AS01 and AS03 incorporated in several new and existing vaccines.

It would take a significant investment to make such a change of adjuvant from existing vaccines. The vaccine company would have to buy rights for using the new adjuvant from the party that owns the IP rights. Also, including a new adjuvant to an existing vaccine will require regulatory approvals as adjuvants are only approved as part of a vaccine, adding another layer of substantial cost. It may also require that changes be made in the manufacturing processes. These enormous costs alone make it extremely challenging for new adjuvants to penetrate the vaccine market.

While we have gained knowledge in how adjuvants work, there are still gaps in our understanding of the MOA of adjuvants. Many factors are bound to affect how adjuvants mediate their immunomodulatory effects. We now know that adjuvant activity can vary remarkably depending on the animal species. For example, CpG ODN used alone is a strong adjuvant in mice and chicken, but is a poor adjuvant in large animals, unless it is used in combination with other adjuvants (reviewed in Mutwiri et al., 2011). The reasons for these remarkable species differences remains unknown.

It should be noted that immunostimulatory adjuvant work by activating innate immune responses which constitute an inflammation response, which if excessive can be deleterious to the host. Thus, too much inflammation will cause pathology and disease, while too little or none may result in lack of adjuvant activity. However, there is no evidence to support this notion. Then the question arises, what is the relationship between degree or nature of the innate immune responses and adjuvant activity? The answer to this question will be valuable in the development of effective and safe adjuvants.

Furthermore, in MOA studies adjuvants are typically used alone with no antigen (Supplementary Table 2), and essentially no studies have explored the possibility that the nature or composition of antigen may influence whether or not a substance has adjuvant activity. The question that we rarely ask is: what is the contribution of the antigen in the overall adjuvant activity of a vaccine? Vaccines typically comprise of antigen + adjuvant, and vaccine antigens can vary in origin and composition. Un-purified microbial antigens may contain components such as CpG DNA or LPS that have known adjuvant activity. In this regard, the resulting antigen-specific immune responses seen in vaccinated animals may be a result of a combination of adjuvant activity from the combination of adjuvant and antigen, such as seen with polyphosphazene plus CpG ODN discussed earlier (Mutwiri et al., 2008). This may explain why a particular adjuvant may promote antigen-specific immune responses in some antigens and not in others. This is a critical piece of information in the rational design of adjuvant+antigen pairing for effective vaccines.

## Conclusion

Over the last decade, evidence has accumulated that polyphosphazenes are potent adjuvants for viral and bacterial vaccine candidates in several animal species. Furthermore, they are effective given by the various routes, and in adults and neonatal animals. These experimental adjuvants are relatively safe, although systematic safety studies are needed. Going forward, there is sufficient pre-clinical data with the newer, more potent polyphosphazene PCEP and its combinations to warrant clinical trials in animals.

## Author Contributions

DC reviewed literature and Prepared first draft of manuscript. GM primary author with overall oversight of the manuscript, conceived idea, checked accuracy of contributions in the manuscript, and edited manuscript. HW contributed material on medical applications of polyphosphazenes. RM contributed material on combination adjuvants. All authors contributed to the article and approved the submitted version.

## Conflict of Interest

GM holds a patent in polyphosphazene adjuvants and their combinations. The remaining authors declare that the research was conducted in the absence of any commercial or financial relationships that could be construed as a potential conflict of interest.
