# Supplementary material for: Polyphosphazenes as Adjuvants for Animal Vaccines and Other Medical Applications
Source: Front Bioeng Biotechnol. 2021 Mar 4;9:625482. doi: 10.3389/fbioe.2021.625482 (PMC7982900; doi:10.3389/fbioe.2021.625482)
Supplement: Supplementary file 1 [file Table_1.pdf]

Supplementary Material

Table 1: Antigen-specific immune responses in animals vaccinated with polyphosphazene adjuvants

Animal species	Adjuvant	Antigen	Route of administration	Immune responses	References
Mice	PCPP	EDIM	IM	↑ neutralizing serum antibody, ↓ viral shedding	McNeal MM 1999
		Influenza virus X:31	SC	↑ serum IgG, dependent on the presence of -COOH groups	Andrianov 2004
		BSA	SC	Significant ↑ total IgG titers	Andrianov 2005
		PR8, PspA, PTd	IN	↑ antigen specific IgA in mucosal secretions for all, ↑ antibody response (IgG1 and IgG2a), vaccine induced antigen specific B cell responses systemically + mucosally, Th1/Th2 cytokine response, all protected against challenge	Shim 2010
	PCPP+CpG	OVA	SC	↑ antigen specific IgG, ↑ antibody response (IgG1, IgG2a), MP formulation showed several fold increase in OVA/CpG in dLNs, MP formulation > soluble, predominantly Th1 response	Garlapati 2010

Mice	PCEP/PCPP	HBsAg and X:31 Influenza virus	IM	PCEP titers 10x > than PCPP, PCEP ↑IgG > PCPP for both antigens	Andrianov 2006
		BSA/X:31 Influenza Virus	SC	↑ total IgG, IgG1, and IgG2a titers where PCEP > PCPP, PCPP primarily IL-4 response whereas PCEP was both IFN-ɣ & IL-4, dose sparing observed with both adjuvants	Mutwiri 2007
		HBsAg	SC	PCEP > PCPP<br>Increased antibody (IgG1, IgG2a)	Mutwiri 2008
		X:31 Influenza Virus	SC	PCEP IgG2a > PCPP IgG2a, PCEP mixed Th1/Th2 response, neutralizing titers for PCEP > PCPP, PCEP/PCPP saw ↑ total IgG and IgG1	Mutwiri 2009
	PCEP	PTd	SC	Predominantly Th1 response, overall ↑ antibody response (IgG2a)	Gracia 2011
		X:31 Influenza Virus	IN, SC, Oral, IR,	IN/SC adjuvant activity > Oral/IR, ↑IgG1 for IN/SC, ↑IgG2a for IN/SC/Oral, balanced Th1/Th2 response in IN/SC, IN/SC yielded IgG/IgA in nasal secretions	Eng NF 2010

Mice		Recombinant MV-H	SC	↑ antibody (IgG1, IgG2a), ↑ MV-H specific IFN-ɣ/IL-5 in vitro indicating CMI, ↑ neutralization titers, retained immunogenicity after 2 weeks	Liubov M. 2012
	PP/PP+CpG	FI-BRSV	IN	↑ humoral response (IgG), PP+CpG > PP alone, significant IgA titers induced, ↑ mucosal IgG and IgA	Mapletoft JW 2008
		OVA	IN/SC	↑ antibody response (IgG1, IgG2a), IgG2a > IgG1, ↑ IgA, ↓ viral replication, IN > SC	Mapletoft JW 2010
	PCEP+CpG+Indolicidin	BRSV - recombinant 'F' protein	IN	↑ antibody response (IgG1, IgG2a), Type 1 CMI, mixed Th1/Th2 antibody response, ↑ cytokine response: IL-12p70, TNF-α, IL-6	Kovacs-Nolan 2009
		BRSV - recombinant 'F' protein	SC	↑ antibody response (IgG1, IgG2a), Th1 or mixed response IgG2a > IgG1 ↑ IFN-ɣ in lungs, ↓ viral RNA	Kovacs-Nolan 2009
	PP4*/PP4+Indol PP4+CpG PP4+CpG+Dye PP4+CpG+Indol	OVA	SC	↑ IgG titers where triple combo > double > single, PP4 at high dose (50 µg) induced tissue damage at SOI, CpG+Indol+PP4 strong Th1, Indol and/or PP4 promote Th2 response	Wilson 2010
	PCEP+CpG+IDR	PTd	SC	↑ PTd specific IgA and IgG, predominantly Th1 response (Il-17, IFN-ɣ), ↑ Th1/Th17 cytokines	Garlapati 2011

Mice	Tri-Adj	PTd	IN	↑ antibody response (IgG2a, IgG1), ↑ duration of immunity in adjuvants formulated with 1:2:1 ratio	Gracia 2011
		RSV-F	IN	↑ affinity maturation of antigen specific IgG, IgA, ↑ VN Abs, ↑ F protein specific CD8+ T cells in lungs, ↑ CD8+ central memory T cells in lymph nodes, enhanced antigen uptake by DCs and trafficking to lymph nodes, observed efficacy and safety profile	Garg 2014
		RSV-F	IN URT/LRT	↑ mucosal IgA, ↑ frequency of IgA-secreting memory B cells, ↑ GC B cells, ↑ Tfh cells, and ↑ plasma cells, upregulated gene expression of AID, IL-6, and IL-21	Garg 2016
		RSV-F	IN	↑ cytokines, chemokines, and interferons in nasal/lung tissues, ↑ Monocyte/DCs recruitment, ↑ antibody response (IgG1 and IgG2a), complete protection in lungs against RSV	Sarkar 2016
		RSV-F	IN	Robust systemic and mucosal response; ↑ antibody response (SIgA, IgG1, IgG2a), ↑ production of IFN-ɣ secreting cells and antigen specific CD8+ T cells, upon challenge no viral replication in lungs, duration of immunity	Garg 2017

Mice				observed to be > 5 months, complete protection against challenge	
	Tri-Adj/L-Tri Adj<br>-Normal Tri-Adj<br>-Lipidic Tri Adj<br>-MP Tri Adj	OVA	IU	↑ antibody response (IgG2a, IgG1*), ↑IgG and IgA, dose sparing with L-Tri-Adj, ↑ IFN-ɣ from lymphocytes for MP/L-Tri Adj, balanced Th1/Th2, L-Tri Adj > Tri-Adj or MP Tri-Adj	Wasan 2019
	Tri-Adj /PCEP	Inactivated PPV, BEI-inactivatedPPV + rPEDV+FliC	IU	No effect on piglet viability or semen functionality, Tri-Adj directly stimulated CCL2/IFN- /CCL4 in UECs, recombinant vaccines failed to elicit humoral immune response	Hamonic 2020
Pigs	PCPP	HBsAg	ID/IM	↑ antibody (IgG titers), dose sparing with ID route, ID formulation > IM	Andrianov 2009
		OmlA from APP	SC	↑antibody (IgG1, IgG2a), ↑IFN-ɣ, humoral efficacy = emulsigen	Dar 2012
	PCEP	OVA	Oral + IP boost	Significant anti-OVA IgM/IgG, IgG1/ IgG2 in serum (5 mg dose), responses observed not simply due to i.p. Booster, piglets respond to systemic antigen exposure with oral tolerance	Pasternak 2014
		SIV	ID/IM	↑ serum antibody, ↑ neutralizing titer, reduced reactogenicity, ↓ viral load in lungs, ID > IM humorally/neutralization	Magiri 2018

		SIV H1N1-A	ID	↑ antigen specific antibodies against H1N1, ↑ IL-13/IL-17A/IFN-ɣ in dLNs, ↑ neutralizing antibodies, ↑H3N2 antibodies compared to control, failed to protect against heterologous challenge	Magiri 2020
Cattle	PCEP+CpG+Indol	HEL	SC	↑ HEL specific IgG antibody titers, long lasting titers, ↑IFN-ɣ, ↑TNF-α, ↑IFN-α	Kovacs-Nolan 2009
	Tri-Adj	BVDV type 2 E2 protein	IM	Significant ↑ E2-specific VN Abs, ↑ IFN-γ, ↑ CD4+/CD8+; as well as increased expression of marker CD25+ indicating CTLs, protection conferred against virulent BVDV-2 challenge	Snider 2014
			IM	Significant ↑ E2-specific Abs, ↑ IFN-ɣ, ↑ CD4+/CD8+ T cells, ↑ CD25+ activation marker indicating CTL production	Snider 2017
			ID	Robust VN antibody response, ↑ CD4+/CD8+ T Cell response, ↑ APCs/DCs, upon virulent challenge no clinical sickness, leukopenia, or viral shedding	Sadat 2017

Rabbit	PCEP+PIC+HDP	OVA/tGD/[rVP2+TrX]	IM<br>IU<br>(Uterus<br>+<br>Lungs<br>+<br>Vagina)	↑ IgG and IgA total serum titers for both OVA and tGD antigens, rVP2+TrX saw significant IgG for IM route only, tGD showed ↑ mucosal IgG only, antigen specific response observed locally and distally (lungs), antigen specific response; OVA/tGD > vP2+TrX	Pasternak 2017
		OVA/tGD	IU	↑IgG antibody response, prolonged protection, dose dependent IgA detected in med/high groups	Pasternak 2018
Ferrets	PCPP	HA H5N1	IM	Dose sparing observed, ↑ stability of vaccine formulations, ↑ survival rate	Andrianov 2011
Lambs	Tri-Adj	BVDV E2 protein	IM	Significant ↑ in E2 specific IFN-ɣ specific cells in blood, protection against virulent BVDV-2 challenge, negligible clinical sickness observed, Tri-Adj formulated with IDR > P:I:C	Snider 2017
		RSV-F + HN of PIV3	IM	↑ antigen specific titers, ewes developed neutralizing titers against PIV/RSV, Lambs had significant neutralizing titers, ↓ PIV lung titers, effective maternal antibody transfer	Garg 2019
Humans	PCPP	HA Influenza	IM	No AEs, efficacy in young > elderly population, sero-protection observed at high doses	Le Cam NB 1998
		HIV-1	IM	Lower risk of AEs with PCPP adjuvanted vaccine	Gilbert 2003

Chicken	PCEP+ABD	IBHV	IM	↑ IgG antibody response, ↑ IFN-ɣ, ↑ IL-12 (p40), ↑ Il-6, balanced Th1/Th2 immune response	Dar 2015
	PCEP+ABD2	FAdV serotype 8b	In ovo	↑ IgG serum titers, KV+PCEP+ABD2 formulation showed highest IgG antibody titers compared to others, upon virulent challenge all formulations conferred protection	Sarfraz 2017
Mice & Pigs	PP+CpG+IDR	PTd+FHA	SC/IM	↑ IgG2a and IgA titers, more balanced Th-1/Th-2 response compared to commercial vaccine Quadarcel, conferred protection against challenge in presence of MatAbs	Polewicz 2013

**Abbreviation**	**Description**
PCEP	Poly[di(sodiumcarboxylatoethylphenoxy)phosphazene]
PCPP	Poly [di(carboxylatophenoxy)phosphazene]
SOI	Site of injection
AEs	Adverse reactions/events
VN	Virus Neutralization

Abs	Antibodies
Ag	Antigen
IN	Intranasal
IM	Intramuscular
IU	Intrauterine
IR	Intrarectal
SC	Subcutaneous
SIV	Swine Influenza Virus
BRSV	Bovine Respiratory Syncytial Virus
MV-H	Measles Virus hemagglutinin
FI-BRSV	Formalin inactivated bovine respiratory syncytial virus
RSV-F	respiratory syncytial virus ‘F’ protein
PPV	Porcine parvovirus
APP	Actinobacillus pleuropneumoniae
HN	Haemagglutinin-neuraminidase
PIV3	Pan-influenza virus 3

BSA	Bovine serum albumin
PspA	Pneumococcal surface protein A
PTd	Pertussis toxoid
HBsAg	Hepatitis B Surface Antigen
ABD	Avian beta defensin
ABD2	Avian beta defensin 2
rVP2+TrX	Porcine parvovirus VP2 and bacterial thioredoxin
tGD	Truncated glycoprotein D from recombinant bovine herpes virus
FAdV	Fowl adenovirus
IBHV	Inclusion body herpes virus
rPEDV	Recombinant porcine epidemic diarrheal virus
FliC	Recombinant Lawsonia intracellularis
BVDV	Bovine viral diarrhea virus
URT	Upper respiratory tract
LRT	Lower respiratory tract
MP	Micro particle

IDR	Innate defence regulatory peptides
Tfh cells	Cells which maintain and help with proliferation of GCs, B cells, and development of plasma cells
MatAbs	Maternal Antibodies
FHA	Filamentous hemagglutinin
KV	Killed virus
EDIM	Murine inactivated rotavirus
PP	Polyphosphazene
PP4	poly(p-dicarboxylatophenoxy)-phosphazene
PP4*	PZ- antigen labelled with infrared dye
HEL	Hen Egg Lysozyme
