# Supplementary material for: Polyphosphazenes as Adjuvants for Animal Vaccines and Other Medical Applications
Source: Front Bioeng Biotechnol. 2021 Mar 4;9:625482. doi: 10.3389/fbioe.2021.625482 (PMC7982900; doi:10.3389/fbioe.2021.625482)
Supplement: Supplementary file 2 [file Table_2.pdf]

# *Supplementary Material*

**Table 2:** Mechanisms of action of Polyphosphazene adjuvants: Non-specific immune responses to polyphosphazene adjuvants injected in the absence of antigen

| Animal species | Adjuvant(s) | Route of administration | Immune responses | References |
|---|---|---|---|---|
| Mice | PCEP | IM | ↑ IFN-ɣ, ↑ inflammasome activation | Awate 2012 |
| | | IM | Dose dependent, pro-IL-1  induction leading to ↑IL-1  /IL-18, ↑ CD8+ T cell response | Awate 2014 (a) |
| | | IM | ↑T cell recruitment to dLNs (CD4/CD8+), ↑ total leukocyte recruitment @ SOI | Awate 2014 (b) |
| | PCEP+IDR+Poly: I:C "P-I-P" | IN | ↑ mRNA and protein expression cyto/chemokines in lung milieu (CXCL10, CCL2, CCL3, CXCL2, CXCL1), ↑ upregulation of TNF-α, IL-1  , and IL-6, ↑ immune cell infiltration observed in lungs without causing toxicity, P-I-P less effective when given further away from challenge- survival rate ↓ | Martinez 2016 |
| | | IN | 100% survival rate upon virulent PVM-15 challenge, ↑ protein concentrations of TNF-α in BALF, ↑ IL-17E in lungs, ↑ AM in response to PIP treatment, innate protection observed to last up to 1 wk | Martinez 2019 |

| | | | | |
|---|---|---|---|---|
| Pigs | PCEP | ID<br><br>ID | ↑Th2 response (IL-6/IL-13), early onset of immune response<br><br>↑ Th2 cytokine response at SOI and dLNs, induce acute inflammation, local inflammation at SOI, trigger innate immunity | Magiri 2016<br><br>Magiri 2019 |
| Chicken | PCPP+CpG-ODN/ PCEP+CpG-ODN | In ovo (SC) | No AEs or poor hatchability in embryos, effective against *E. coli* challenge, significant ↓ in mortality in embryos immunized with PCPP/PCEP+CpG-ODN compared to control; PCEP alone also provided modest protection | Taghavi 2009 |

| **Abbreviation** | **Description** |
|---|---|
| AEs | Adverse reactions/events |
| PCEP | Poly[di(sodiumcarboxylatoethylphenoxy)phosphazene] |
| PCPP | Poly [di(carboxylatophenoxy)phosphazene] |
| SOI | Site of injection |

| | |
|---|---|
| IN | Intranasal |
| IM | Intramuscular |
| ID | Intradermal |
| SC | Subcutaneous |
| MP | Micro particle |
| BALF | Bronchoalveolar lavage fluid |
| dLNs | Draining lymph nodes |
| IDR | Innate defense regulatory peptides |
| MatAbs | Maternal Antibodies |
| PVM-15 | Pneumonia virus of mice strain 15 |
